# Extrusive Luxation Injuries in Young Patients: A Retrospective Study with 5-Year Follow-Up

**DOI:** 10.3390/dj8040136

**Published:** 2020-12-16

**Authors:** Enrico Spinas, Laura Pipi, Claudia Dettori

**Affiliations:** 1 Department of Surgical Sciences, Sports Dental Research Center, University of Cagliari, Via Ospedale, 40-09124 Cagliari, Italy; enricospinas@tiscali.it; 2 Department of Conservative Dentistry and Endodontics, School of Dentistry, University of Cagliari, Via Ospedale, 40-09124 Cagliari, Italy; claudia.dettori@gmail.com

**Keywords:** dental trauma, extrusion, pulp canal obliteration, permanent tooth, pulp necrosis

## Abstract

(1) Background: The purpose of this study was to analyze the influence of the chosen diagnostic and therapeutic approach (repositioning and splinting methods) on the risk, frequency and timing of the onset of pulp canal obliteration and pulp necrosis following extrusive luxation in young patients with permanent dentition. (2) Methods: From an initial sample of 50 subjects affected by extrusive luxation, were selected the clinical data of 13 patients presenting extrusive luxation but no other type of injury to the dental hard tissue. All teeth were examined according to a standardized protocol. Follow-up examinations were performed at regular intervals for 5 years. Statistical associations between pulp consequences and several covariates were assessed using the Mann–Whitney test and Fisher’s exact test. (3) Results: Among the 13 studied teeth, only 1 healed completely, whereas 9 showed pulp obliteration and 3 developed pulp necrosis. No tooth with obliteration developed pulp necrosis. The average time to treatment was 11.9 h. The treatment approaches used were manual repositioning, orthodontic repositioning and stabilization splinting. “Time to treatment” was the only covariate that showed a weak statistical association with the onset of pulp consequences. (4) Conclusions: There is still uncertainty over the most appropriate therapeutic approach to adopt in young patients with extrusive luxation injuries, particularly for repositioning of the injured tooth. Extruded teeth should be treated as soon as possible after the traumatic event. This study highlighted the value of orthodontic repositioning of the extruded tooth, which does not seem to aggravate the conditions of the dental pulp. In addition, the study confirmed that prophylactic endodontic treatment is not appropriate for immature teeth affected by extrusive luxation injuries, given the extreme rarity of pulp necrosis in teeth already affected by pulp obliteration.

## 1. Introduction

Traumatic injuries to permanent teeth are common, affecting 10.5–17.3% [1,2] of the population. In 18–33% [3,4] of cases there is damage to pulp and/or supporting tissue. Extrusion, lateral luxation and intrusion are among the most serious types of dental trauma. Few studies have focused specifically on extrusion, which has been reported to account for around 3% of all cases of traumatic dental injury [5].

Extrusive luxation injuries are caused by the action of an oblique force, and they are characterized by high mobility and partial dislocation of the tooth outside its socket [6]. Also termed partial avulsions [5,7], they can disrupt the vascular supply to the pulp [8,9]. Clinically, the tooth appears elongated and often displaced palatally [5]; occlusion and mastication are painful, and spontaneous pain, if present, is only mild [10]. Radiographically, the apical and lateral parts of the socket appear empty [11] and, overall, the periodontal ligament space is enlarged [5,6].

Pulp canal obliteration (PCO) and pulp necrosis (PN) are the most frequent consequences of extrusive luxation, while root resorption (RR) is less frequent [12,13].

Physiological healing (without pulp modifications) is another possible outcome, which occurs more frequently in teeth with open apex [12].

Complications depend on factors such as the severity of the lesion, the stage of root development and the presence of a high oral bacterial load [5,9,14]. Pulp and periodontal complications are more frequent in teeth with fully formed roots as opposed to open apex [9,12,14] and, since they can arise weeks, months or years after the trauma, long-term follow-up is essential [11,13,14]

The prevalence of PCO as a consequence of avulsion and luxation injuries is between 3 and 24% [12,15], while higher rates of PN are reported following dental trauma: 64% after extrusion, 77% after lateral luxation, and up to 100% after avulsion injuries [13,16].

As mentioned, the pulp of teeth with open apex has a high healing potential. In these teeth, the pulp response may evolve towards physiological healing or, more often, PCO [12].

On the contrary, teeth with closed apex are very unlikely to show revascularization and often develop PN [9,13]. In the literature, post-extrusion PN has a prevalence of between 43 and 56% [9,17], and it is considered the most common post-traumatic complication [14,18].

Ideally, extrusive luxation injuries require immediate treatment [10], consisting of repositioning and stabilization. This should be followed by long-term monitoring of the tooth [11,13,14]. This approach allows better control of post-traumatic complications and increases the chances of survival of the tooth and supporting tissues [19].

Treatment begins with a correct clinical and radiographic diagnosis, followed by manual repositioning of the tooth in the socket and placement of a flexible splint, to remain in situ for about 2 weeks [11]. According to the 2020 IADT (International Association of Dental Traumatology) guidelines [11], the splinting time can be prolonged by a further 4 weeks if there is breakdown/fracture of the marginal bone. 

Manual repositioning is often impossible due to the formation of a blood clot in the socket, especially in the case of delayed treatment. In these cases, alternative therapies need to be considered, such as surgical repositioning [20] or orthodontic repositioning (re-intrusion of the tooth) [21].

Any attempt to examine this topic on consistent investigations and meta-analysis of data is hampered by the scarcity of literature focusing specifically on extrusion, and the genesis and timing of the most common pulp consequences (PCs): PN and PCO. Furthermore, there is no univocal therapeutic approach to the management of pulp reactive processes [22], while the current guidelines, deriving from animal studies, case studies and clinical experience, are not based on strong evidence [23]. Given the lack of clinical studies in this field, there are no clear indications on complications, risks, benefits and economic factors [12].

With the aim of adding to the available data on this topic, the authors conducted a retrospective observational clinical investigation in a sample of young subjects with extrusive luxation injuries to permanent teeth. The five-year post-treatment follow-up of the 10 selected patients yielded significant data on various types of pulp response triggered by these dental traumas.

In particular, the following aspects were analyzed: the influence of the initial therapeutic approach (especially the use of the orthodontic splint) on the development of PN and PCO; onset times and frequency of PN and PCO with respect to the severity of the extrusion; the frequency of onset times of PN and PCO with respect to the root maturity of traumatized teeth; the frequency of onset of PN in teeth already affected by PCO.

This study set out to evaluate specifically the relationship between PCs and some covariates of known interest (mm of extrusion, time to treatment, stage of maturation of the apex, type of splinting and crown color changes). The sample was carefully selected to reduce the variability that arises from the concomitance of other pathologies (for example crown and root fractures, subluxations or concussion) and their consequences. The aim was thus to conduct a narrow but detailed analysis focusing on teeth presenting only extrusive luxation injuries.

Due to the small sample size, not all variables considered can be analyzed statistically. Therefore, in some cases only meta-data are reported.

## 2. Materials and Methods 

A retrospective observational study was conducted by first analyzing the records of 50 patients with extrusive luxations in permanent dentition. 

All patients, aged between 8 and 16 years, were visited and treated at the Dental Traumatology Centre at the University of Cagliari (Italy) between 2008 and 2015.

The treatments were conducted by two specialists, equally qualified and with equivalent clinical-diagnostic expertise, who intervened alternately both in the provision of acute treatment and in the subsequent follow-up care.

All patients admitted to the study had suffered extrusive luxation to permanent incisors. A total of 65 teeth were selected: 60 upper incisors and 5 lower incisors.

The sample was then further selected using the following inclusion criteria:

1. Patients who had at least one permanent incisor presenting only extrusive luxation;
2. Patients examined within 48 h of the traumatic event and whose data had been collected according to the IADT protocols of 2007 [24] or 2012 [25];
3. Patients who were followed up continuously for at least 5 years.

The exclusion criteria were as follows:

1. Patients who had suffered a previous trauma before the event and/or presented carious lesions;
2. Patients in whom the trauma caused uncomplicated or complicated lesions to the hard tissue of the extruded teeth;
3. Patients who suffered further traumatic injuries during the follow-up period;
4. Patients with ongoing orthodontic therapy.

Selections were made on clinical and radiographic findings, by two equally qualified and experienced specialists, working individually. All selected cases were checked (focusing especially on their radiographic data) by a third specialist with greater experience, expertise and decision-making authority. The kappa test was run to evaluate the level of agreement between these experts, and it was found to be “moderate” (Landis and Koch, 1977) [26]. Any disagreements were resolved through discussion between all three reviewers.

Application of these strict criteria yielded a final sample of 10 patients (5 males and 5 females), with a total of 13 teeth, all permanent upper central incisors, affected only by extrusive luxation. 

**Figure 1:**
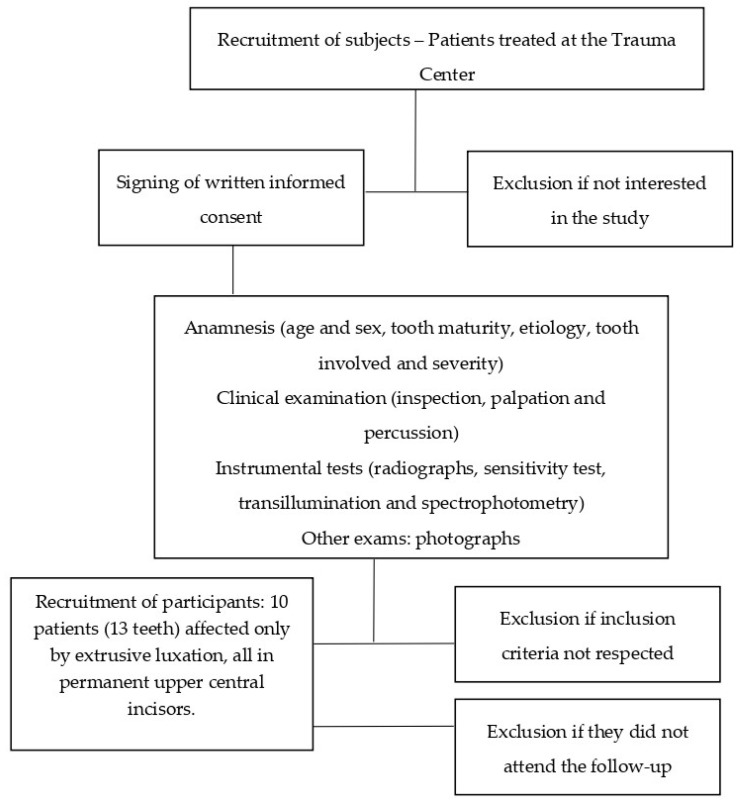
The recruitment process that led to the final sample.

Figure 1 summarizes, as a flow chart, the patient recruitment process. 

### 2.1. Management of Extrusions

The data collection chart reported, as per protocol, the patient’s age and sex, the etiology of trauma, the tooth involved and the type and severity of the injury [6]. In addition, it included a clinical description of the lesion, information on pulp sensitivity test responses, radiographic and photographic investigations, type of treatment and details of the long-term follow-up [27,28]. 

Before the start of the treatment, written consent to the clinical and therapeutic investigations was obtained from the parents/legal guardians of all the patients.

The study was conducted in accordance with the Declaration of Helsinki, and the protocol was approved by the Ethics Committee of Department of Surgical Science at University of Cagliari (Italy) (DSC 001027) on 30 September 2015.

In all cases, patient assessment began with collection of the history, followed by intra- and extra-oral examination and palpation of the injured area. The dental clinical examination included the testing of mobility, sound and tenderness to percussion, and response to pulp tests. At follow-up examinations, in addition to repeating the above, the following aspects were recorded: the presence/absence of fistulae, gingivitis, gingival retraction and gingival sulcus depth [27].

Pulp sensitivity was tested both with a Siemens Sirotest II^®^ electric pulp tester (Siemens, West Germany) and with a cold spray (maximum temperature −26.5 °C). A negative response recorded soon after the trauma was interpreted not as a sign of pulp death, but as a shock index, and therefore as remissible over time.

Radiographs (standardized periapical and occlusal) were obtained at 55 kVp, 15 mA with an exposure time of 1 s using 3 × 4 mm or 2 × 3 mm Kodak film.

Crown color changes were diagnosed by transillumination using a Siemens Silux lamp situated at a distance of about 80 cm from the oral cavity. 

From 2009, patients were also examined using the SpectroShade Micro (MHT, Verona—Italy), which detects even minimal color changes. The Vita Color Scale A, B, C, D (VITA Zahnfabrik. Bad Säckingen, Germany) was used for objective description of tooth crown color.

Extrusive luxation was diagnosed using the 1992–2007 WHO–Andreasen classification system [29].

The stage of root development at the time of injury was established using the classification system introduced by Moorrees [30]. In the present research, teeth with incomplete roots were those with a root length corresponding to one quarter, half, or three quarters of the total tooth length and with open apices (Moorrees Stages 1–5). Teeth with full root length and closed apices were considered completely formed (Moorrees Stage 6). 

**Figure 2:**
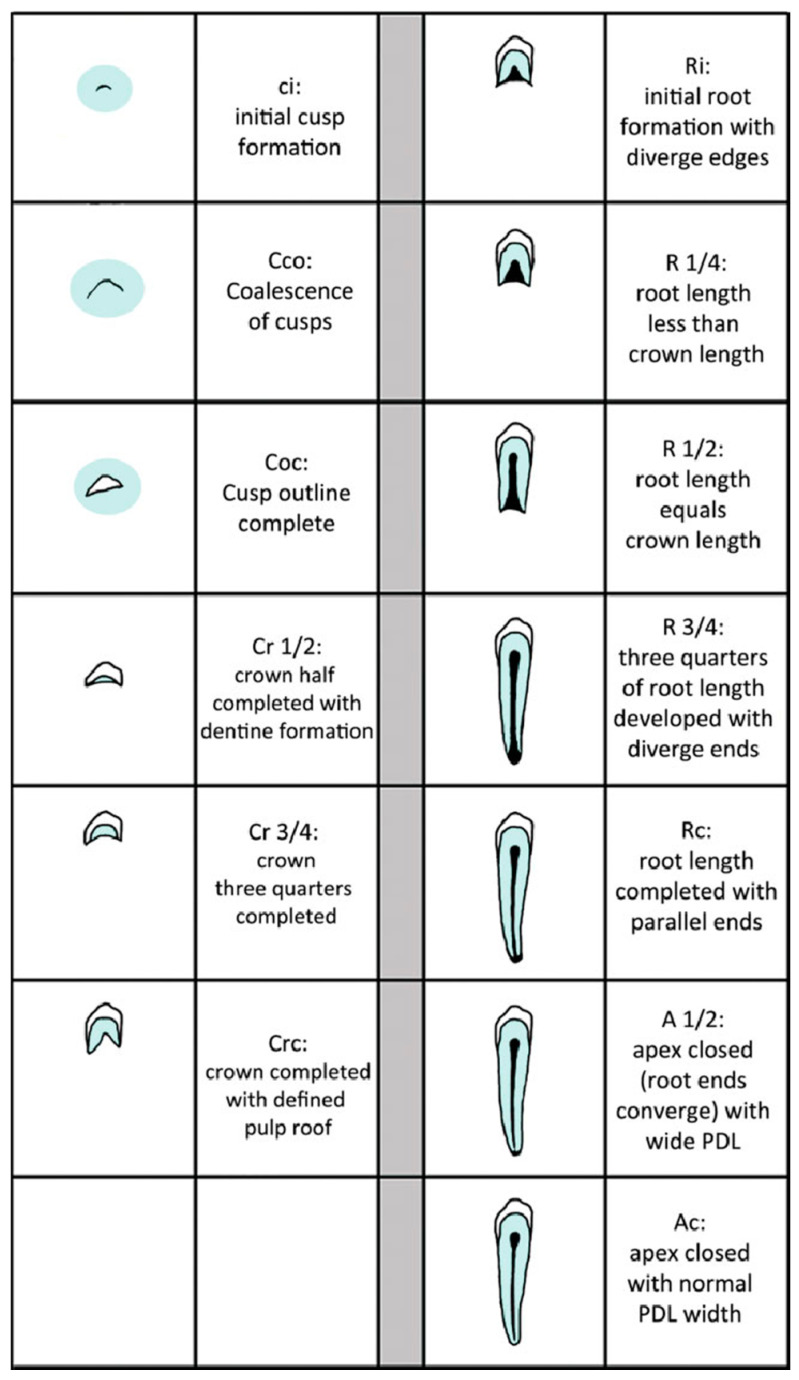
The stages of tooth formation for assessing the development of single-rooted teeth. Taken from S.J. AlQahtani, M.P. Hector, and H.M. Liversidge. The London Atlas of Human Tooth Development and Eruption. Am J physical anthropology 2010, 142(3):481–490.

Figure 2 lists the Moorrees stages, showing the development of cusps and root. 

The degree of extrusion was calculated in millimeters based on radiographic reference points; in particular, the distance from the apical foramen to the base of the alveolar socket was measured [5]. Extrusion was classified as (a) mild, 0–2 mm, (b) moderate, 3–5 mm and (c) severe, ≥6 mm. 

Tooth repositioning was not standardized but managed on a case-by-case basis, and it depended very much on the time that had elapsed since the initial trauma. 

Extrusive luxation is normally treated under local anesthesia [11]. After radiographic assessment, the luxated tooth is repositioned manually and stabilized using a flexible splint [11,24,25]. When manual repositioning is not possible, surgical and orthodontic repositioning are alternative approaches.

Surgical repositioning, or intentional replantation [20], involves the extraction of the extruded tooth followed by irrigation and gentle cleaning of the socket. The tooth is then reinserted into the socket as quickly as possible [31]. Appropriate endodontic treatment needs to be performed within 15 days of the dental trauma to prevent the onset of IRR [32,33].

In the present study, whenever manual repositioning was not possible, the authors used the orthodontic repositioning technique. This was performed using NiTi orthodontic arch wires (0.14/0.16) inserted in preadjusted edgewise orthodontic brackets (0.018” by 0.022”) (Opti-MIM, Cerum Ortho Organizers, Calgary, AB). The wire, generating light forces of up to 40gr, produced a gentle and gradual movement of the extruded tooth in the socket [34].

For greater stability, the orthodontic repositioning splint always included at least two healthy teeth, one on either side of the injured tooth [35]. The repositioning took 30–40 days and was followed by a stabilization period of 14–21 days during which the same orthodontic splint was used.

**Figure 3:**
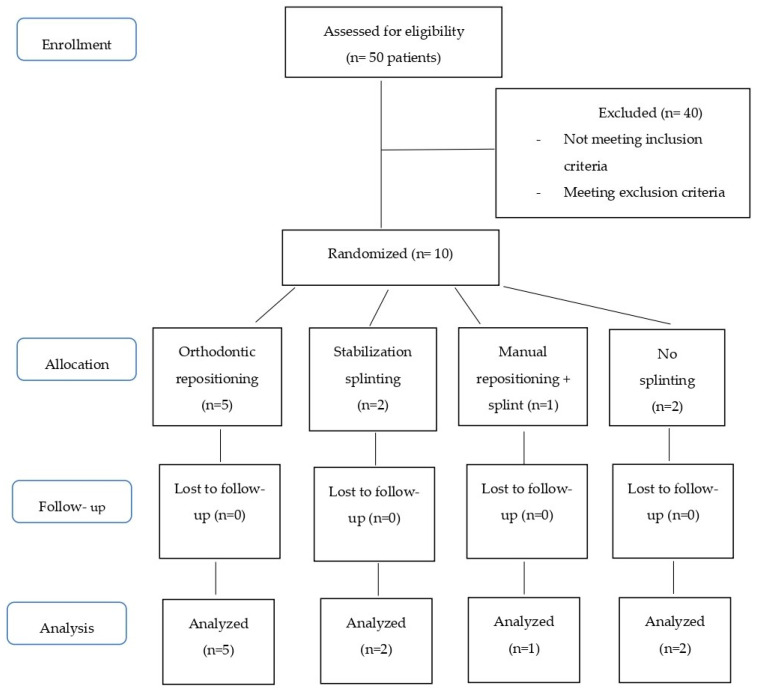
Flow diagram showing the phases of the study: enrollment, allocation, follow-up, analysis.

Long-term follow-up included clinical and radiographic examination at 2 weeks, 4 weeks, 6–8 weeks, 6 months, 1 year and annually thereafter for 5 years, as the IADT [11,24,25] and AAE [36] guidelines suggest. Figure 3 presents, as a flow diagram, the phases of the study. 

The diagnosis of pulp healing, PN, PCO and RR was based on clinical and, in particular, radiographic data, collected at each follow-up visit. Diagnosis of PN was based on the absence of pulp sensitivity, together with other clinical signs—a grey-colored crown, periapical radiolucency on radiographic examination and tenderness to percussion [15]. When PN was detected, appropriate endodontic therapy was carried out.

In the present investigation, the following radiographic criteria were used to establish the diagnosis of partial and total PCO. In partial PCO, the coronal part of the pulp chamber is no longer visible, while the root canal is markedly narrowed, but still visible. In total PCO, the entire pulp canal is barely visible or not visible at all [12]. This radiographic evidence, when associated with a lack of response to electrical and thermal tests and yellow discoloration of the crown, points to full-blown PCO [37].

The diagnosis of IRR (inflammatory root resorption) is usually made on a routine radiography because the condition is often clinically asymptomatic. Radiographically it appears as areas of resorption (radiolucent areas) on the root surface [38].

### 2.2. Statistical Analysis

A statistical analysis was conducted to look for associations between the responses to variable pulp consequences *(PCs)*, in particular, the onset of PN and PCO, and some of the covariates considered in the study (mm of extrusion, time to treatment, stage of maturation of the apices, type of splinting and crown color changes). 

PCO was considered as a para-physiological reaction that only requires constant monitoring as it does not usually give rise to further complications requiring new treatments. PN was considered a pathological outcome with serious implications. The inferential results are reported in Table 1.

Specifically, the authors evaluated the associations between:

- PCs and mm of extrusion;
- PCs and time to treatment (in hours).

In particular, they assessed associations between:

c. PCs and mm of extrusion categorized into three classes (mild, moderate, severe);
d. PCs and stage of maturation of the root apex (open apex (OA) and closed apex (CA));
e. PCs and type of splinting (stabilization, orthodontic repositioning);
f. PCs and crown color changes.

Because of the limited number of observations, a nonparametric approach was chosen. The Mann–Whitney test was used for comparison between means (a. and b.) and the Fisher Exact test for comparisons between categories (c–f). Statistical analyses were performed using STATA 16 software (Stata Corp LLC., College Station, TX, USA). The significance level of all tests was set at *p* < 0.05.

## 3. Results

### 3.1. General Data of the Selected Patients

Table 2 shows the epidemiological and etiological data collected—patient age and sex, the affected tooth, the maturity of the apex and the etiology of the injury. The most frequent cause of trauma was sport (seven teeth), followed by domestic accidents (five teeth). Only one dental extrusion was the result of assault. The patients were aged between 8 and 16 years (six were aged 8–10 years, one was 12 years old and three were between 14 and 16 years old). Gender was equally distributed in the sample—five males and five females. The tooth most frequently involved (*n* = 8) was the upper right central incisor (tooth 11). The other five injured teeth were all upper left central incisors (tooth 21). Of the 10 patients, 3 had two injured teeth. Eight teeth were classified as Moorrees stages 1 to 5 and the other five as Moorrees stage 6.

**Figure 4:**
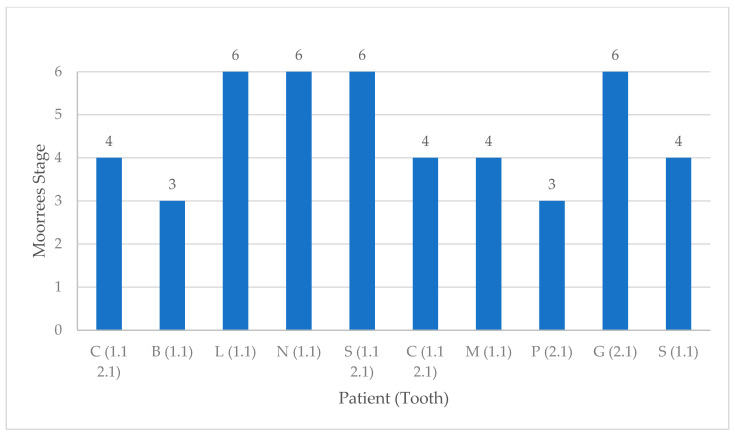
Tooth and root development as assessed at the time of injury using the Moorrees stages.

Figure 4 shows the distribution of patients on the basis of the Moorrees classification.

### 3.2. Severity of the Extrusion 

Extrusion of the tooth at the time of the trauma was rated as absent, mild (0–2 mm), moderate (3–5 mm) or severe (≥6 mm). The sample contained four teeth with mild extrusion, seven with moderate extrusion and two with severe extrusion. No statistically significant association was found between the severity of the extrusion and the PCs (*p* = 0.634; Table 1).

### 3.3. Time to Treatment

Only three teeth were treated within 4 h of the trauma. Seven teeth were treated after a delay of between 8 and 12 h, one tooth after 13–18 h, and two teeth at about 24 h. The average time to treatment in this sample (calculated considering the 10 patients) was 11.9 h. 

A weak statistical relationship was found between time to treatment and different PCs (*p* = 0.060; Table 1).

### 3.4. Orthodontic Repositioning, Splinting and Treatment Time

Of the 13 teeth, 3 were treated by manual repositioning in the socket followed by placement of a stabilization splint (this was done using the acid-etch technique and braided wire).

Two immature teeth were treated only with stabilization splinting; one, with moderate extrusion, was treated after 8 h, and the other, with severe extrusion, was treated 24 h after the trauma. 

All these splints were removed within 3 weeks of placement. 

Seven teeth underwent orthodontic repositioning, a process that lasted 30/40 days on average; these teeth remained splinted for approximately 60 days.

The study sample included four teeth showing mild extrusion—two (one OA and one CA) were treated with orthodontic repositioning, and one was an immature tooth treated with manual repositioning and application of a stabilization splint. The remaining tooth, also with open apex, was treated only with manual repositioning. 

Moderate luxations (seven teeth) were managed with orthodontic repositioning (two OA and two CA), with stabilization splinting (one OA), with manual repositioning and stabilization splinting (one OA) and with manual repositioning (one OA).

Severe luxations (two teeth) were treated with orthodontic repositioning (one CA) and with stabilization splinting (1 OA). 

No statistically significant association was found between type of splinting and PCs (*p* = 0.345; Table 1).

### 3.5. Pulp Consequences 

The sample included nine teeth with PCO and three with PN. The remaining tooth (CA with moderate extrusion treated after 10 h) showed physiological pulp healing.

#### 3.5.1. Pulp Canal Obliteration 

PCO was observed in seven immature and two mature teeth. No statistically significant association was found between stage of apical maturation and PCs (*p* = 0.236). 

PCO occurred in three teeth treated early (<4 h), in five teeth treated between 5 and 12 h after trauma, and in one tooth treated after 19–24 h. 

Cases of PCO were observed with all the types of treatment used—in four teeth following orthodontic repositioning (three OA and one CA), in one tooth after stabilization splinting, in two teeth (OA) following manual repositioning and stabilization and in two non-splinted teeth (one manually repositioned mature tooth and one immature tooth which did not undergo repositioning).

PCO occurred in three teeth with mild extrusion (about 2 mm), in five teeth with moderate extrusion and in one tooth with severe extrusion. No statistical association was found between severity of extrusion and PCs (*p* = 0.727; Table 1).

Finally, none of the nine teeth with PCO (seven OA and two CA) showed an evolution of PCO towards PN, either during or at the end of the follow-up (Table 3). In this case, no statistical analysis was carried out, as this outcome, not being observed, could not be compared with the other parameters analyzed in the research.

#### 3.5.2. Pulp Necrosis

PN occurred in three teeth (one OA and two CA), treated after delays of 5–12 h, 13–18 h and 19–24 h, respectively.

During the study, PN developed in two teeth with CA treated with orthodontic repositioning and in one tooth with OA treated with stabilization splinting. In the latter case, it appeared within the first year of the traumatic event, and then evolved into IRR after 3 years. 

PN was equally distributed in relation to extrusion severity, being found in mild (one tooth), moderate (one tooth), and severe (one tooth) extrusion.

### 3.6. Pulp Sensitivity and Crown Color Changes

Five teeth showed no response upon pulp sensitivity testing immediately after the trauma—four of these regained sensitivity after a few months, although it remained decreased for the first year, but was lost completely after 3 years due to onset of PCO. In the other tooth, which developed PN, sensitivity was never regained.

Seven teeth conserved mild sensitivity after the traumatic event. Of these, two later developed PN and lost sensitivity within the first year, while five developed PCO, which became clinically detectable about a year after the trauma. In these latter cases, pulp sensitivity remained reduced for the first year, but was permanently lost 3 years post-trauma due to the PCO.

Only one tooth healed and remained sensitive both immediately after the trauma and subsequently. This was a closed apex tooth showing moderate extrusion and it was managed through orthodontic repositioning 10 h after the trauma.

Crown color changed in 12 of the 13 analyzed teeth—9 of these, all with PCO, turned yellow. This was noted, respectively, within the first year of the trauma (two teeth) or 1–2 years (three teeth), 2 years (three teeth) or 3 years (one tooth) afterwards. Three teeth turned grey due to PN—in two teeth within the first year of the trauma and in the remaining tooth 3 years after the event.

No statistically significant association was found between crown color changes and PCs (*p* = 0.318; Table 1).

### 3.7. Further Dental Traumas and Orthodontic Therapies

No patient experienced any further dental trauma during the follow-up. One patient with an OA tooth and severe extrusive luxation (treated 24 h post-trauma) started orthodontic treatment 1 year after the trauma and, in the meantime, developed PN, which, at 3 years follow-up, was found to have evolved into IRR.

Table 3 summarizes the anamnestic information collected, treatments performed and clinical PCs, reported in more detail below.

## 4. Discussion

The etiological factors found in this sample were comparable with those most frequently reported in the literature. Sports accidents, road accidents, falls, domestic accidents and, increasingly frequently, assault are the most common causes of traumatic dental injuries [1,2,14,39]

According to the literature, the teeth most frequently involved in traumatic dental injury are the upper central incisors, followed by the upper lateral incisors, while the lower ones are less frequently affected [18,40]. Our sample is perfectly in line with these findings as all the analyzed teeth were central incisors.

Many cases of dental trauma involve a single tooth; however, multiple injuries are also reported [2]. The present sample included three patients with traumatic extrusive luxation injuries to two teeth simultaneously.

Root maturity is the key factor in pulp healing after extrusion injuries. In teeth with open apex, the pulp can heal physiologically or PCO can occur. In teeth with closed apex the probability of PCO is lower and PN often occurs [12]. The presence of bacteria in the root canal is the main cause of pulp revascularization failure [9]. Once PN is diagnosed, appropriate endodontic therapy must be started to eliminate the infection and facilitate healing.

On the basis of the overall diagnostic findings in our sample, it is possible to advance some considerations with regard to the main aspects analyzed in this research. 

### 4.1. The Influence of the Initial Therapeutic Approach (Especially the Use of the Orthodontic Splint) on the Development of PN and PCO

There are three ways of treating extrusive luxation injuries, namely through manual, orthodontic or surgical repositioning. In 2002, Andreasen et al. [10] argued that the choice of treatment should also depend on its timing in relation to the injury. Accordingly, they distinguished between acute (within the first 3 h), subacute (within 24 h) and delayed (after more than 24 h) treatment. 

Albeit in a small sample of patients, this study showed a weak statistical association (*p* = 0.060) between the PCs manifested and the timing of the treatments. 

Acute treatment (within 3 h) was not possible in any of our patients—all were treated between 3 and 24 h after the trauma. 

Three teeth were treated using manual repositioning and all these teeth developed PCO. This approach was used for moderate–mild extrusions with a maximum dislocation of 3 mm. 

However, when manual repositioning cannot be used, as in the case of delayed treatment, it is necessary to use other strategies, such as orthodontic repositioning. This is a slow, gradual process that can help to safeguard dental pulp vitality, prevent ankylosis and promote periodontal healing, especially in healthy young subjects [41,42]. 

In the present study, the treatment was chosen on the basis of the time that had elapsed since the trauma, the severity of the extrusion and the need to quickly eliminate the dental interference (i.e., occlusal trauma or OT) in order to restore a correct occlusal relationship [43]. Dental interference, if maintained, causes serious hypofunction. Accordingly, orthodontic repositioning, which very often consists of a vestibular intrusion movement to reposition palatally extruded teeth, was chosen for seven luxated teeth.

After orthodontic repositioning, four teeth developed PCO (three OA and one CA) and two PN (both CA). One tooth (CA) showed physiological pulp healing. 

Table 4 analyzes the PCs in relation to mm of extrusion, type of splinting and time to treatment. 

To avoid the increased risk of IRR [32], no teeth in the sample were treated with surgical repositioning. Only 3 cases of PN and 1 of IRR were recorded (among the 13 analyzed teeth), from which it might be assumed that the controlled orthodontic repositioning technique prevented further injuries at the intra-alveolar and apical level [43]. 

Table 5 summarizes the most recent information regarding orthodontic repositioning treatment [21,42,44]. The approach adopted by Ebrahim and Kulkarni [42], which was similar to ours, allowed these authors to obtain physiological healing and the continuation of root development once the occlusal interference had been removed, even if the tooth was repositioned 14 days after trauma.

Despite some differences between the three case reports summarized in Table 5, in all cases the treatment led to conservation of the extruded tooth without radiographic signs of periapical bone disease or RR. There are still few studies in the literature that investigate the possible onset of sequelae in traumatized teeth subjected to orthodontic tooth movement. The most appropriate timing of orthodontic treatment after trauma remains an open question.

To date, there is insufficient evidence of an increased risk of PN in previously traumatized teeth undergoing orthodontic tooth movement, compared with those without previous dental trauma [45]. 

Moreover, although Amaral et al. [23] found no general consensus on the use of orthodontic movements after severe luxation, it is assumed that therapeutic orthodontic treatment can be resumed, in the absence of ankylosis, 6 and to 12 months after the trauma. However, doubts and contradictions remain.

Finally, the management of therapeutic orthodontic treatment in patients with a history of delayed treatment of dental trauma remains poorly understood. Van Gorp et al. [46] in 2020 and Sandler et al. [47] in 2019 revealed significant gaps in the knowledge of how to manage orthodontic treatment after dental trauma. Sandler et al. [47] highlighted the need for additional training and national guidelines, while Van Gorp et al. [46] called for more specialized training.

### 4.2. Onset Times and Frequency of PN and PCO with Respect to the Severity of the Extrusion

PN is a frequent post-traumatic manifestation with a higher prevalence in teeth with complete root maturation as opposed to immature roots [9,13]. PN, when it arises after an extrusion injury, often occurs within the first year of the traumatic event [13,18]. In the current study, PN occurred 6 months after the trauma in a tooth with open apex and mild extrusion, and within the first year in two teeth—respectively a severely extruded tooth with open apex and a moderately extruded tooth with closed apex. PCO is a frequent consequence of extrusion, as there is less neurovascular damage after extrusion compared with intrusion and avulsion injuries [5]. Detectable radiographically as a narrowing of the pulp chamber and canal, the PCO process begins about a year after the traumatic event and is completed within the five years following the trauma [12]. In this regard, the pattern observed in our sample was completely in line with what has been found in the literature.

Table 4 shows that PCO was more frequent in moderate (five teeth) and mild (three teeth) extrusion, but was found in only one tooth affected by severe extrusion.

### 4.3. Frequency of Onset Times of PN and PCO with Respect to the Root Maturity of Traumatized Teeth

The stage of apical maturation is certainly the key factor in pulp healing after extrusion. The pulp of teeth with OA has great healing potential, which can promote physiological healing or PCO. Teeth with CA, on the other hand, are unlikely to develop PCO and PN often occurs [12]. 

Table 6 shows data on the frequency of occurrence of PN and PCO, published by Andreasen et al. [12], Lee et al. [17] and Hecova et al. [14]. 

The combined data from three studies (Andreasen et al. [12], Lee et al. [17], Hecova et al. [14]) concerning 191 teeth repositioned after extrusions, of which not all parameters have been indicated (e.g., time from trauma, severity of luxation), showed 115 teeth with pulp involvement. Of these teeth, 53 (27.74% of the total) developed PCO and 62 (32.46%) PN (Table 6). 

They reported 53 teeth with PCO, of which 40 had OA (75.47%) and 13 CA (24.53%); of the total 62 teeth showing PN, 15 had OA (24.19%) and 47 CA (75.81%). In the current sample of 13 extruded teeth, 12 teeth were found to be affected by PCO or PN, and only one tooth showed physiological healing. 

Of the 13 analyzed teeth, 69% showed PCO, and a far greater proportion than that (3 teeth, just 23%) developed PN. 

Specifically, nine teeth showed PCO (seven OA and two CA), and three PN (one OA and two CA). In percentage terms, 69.23% of our sample showed PCO (77.77% immature and 22.23% mature teeth) and 23.07% PN (33.34% immature and 66.66% mature)—rates very similar to those recorded by the above authors.

Concerning the selection of the sample, we would like to point out that of the three authors, only Lee et al. [17] specified that patients with concomitant injuries to hard tissue had been excluded, while Andreasen et al. [12] made no mention of such patients. The result reported by Hecova et al. [14], on the other hand, include data on 21 luxated teeth with concomitant crown fracture, but without specifying the type of luxation injuries in which this was observed. This information would actually be relevant, given that this author reports a 66.6% increase in the onset of PN, while Lauridsen et al. in 2012 [9] reported an increase from 56% to 76% in the onset of PN, in teeth presenting concomitant injuries.

Our data, albeit referring to a small sample, confirm what has been reported in the literature [12,14,17], namely that the healing response of teeth following extrusive luxation depends very much on the stage of root development and the severity of the dislocation. 

Physiological pulp healing was found in only 1 (CA) of the 13 teeth. In this regard, only Andreasen et al. [12] reported data comparable to those reported herein; analyzing 48 teeth, they found 13 (10 OA and 3 CA) with pulp survival (27.48%). On the other hand, Hecova et al. [14] reported physiological healing in 49 (48 CA and 1 OA) out of 89 teeth after extrusive luxation (55.05%). These data seem to need further investigation, particularly given the very low rate of teeth with PCO (12 of the 89 teeth, including only 5 with OA).

### 4.4. Frequency of Onset of PN in Teeth already Affected by PCO

A diagnosis of PN after PCO, to be certain, must be based not only on lack of response to pulp sensitivity tests (an obvious and progressive consequence of pulp lumen reduction), but also on the finding of clear apical radiolucency on X-ray examination associated with a change in crown color [37]. Although this topic is widely discussed in the literature, inadequate diagnostic approaches continue to prompt unmotivated clinical-therapeutic choices that can often result in serious biological damage.

To illustrate this point, prophylactic endodontic treatment is contraindicated in teeth that are developing PCO, as it exposes the patient to a risk of serious iatrogenic errors (e.g., deviations or false canals, perforations and instrument fractures in the canal lumen), which drastically worsen the prognosis of the tooth involved [22,48]. 

The development of PN following PCO is uncommon (reported in between 1 and 27% of cases) [48]. PCO may evolve into PN in the presence of impaired vascular supply to the pulp. For example, orthodontic movement, especially intrusive, and new traumas may increase the risk of PN if the pulp lumen is already narrowed [49,50,51,52]

Into Table 6 are summarized the data on the development of PN after PCO. Drawn from the papers of Andreasen et al. [12], Lee et al. [17], and Hecova et al. [14], these data confirm the extreme rarity of this complication. Andreasen et al. [12] report a single case out of a total of 48 (approx. 2%), while Lee et al. [17] report 2 cases among 55 extruded teeth (approx. 3.6%). Hecova et al. [14] do not refer to these data.

In the current study, none of the teeth with PCO developed necrosis and the obliteration process was completed (within 5 years) without any worsening of the pulp conditions. This finding could not be analyzed statistically in the present study: since no case of PN after PCO was reported, it was not possible to compare this pulp outcome with the other analyzed covariates.

Various authors [48,53,54,55] confirm that root canal treatment in a previously traumatized tooth should be performed only in the presence of primary PN, a diagnosis that must be based not only on negative pulp tests, but also on clear clinical and radiographic findings.

## 5. Conclusions

Numerous doubts persist in the literature on the correct therapeutic approach to adopt in cases of traumatic extrusive luxation in young patients. Despite the small size of the sample, this retrospective study provides useful indications for identifying biological or therapeutic factors that might predict a pathological (PN) or para-physiological (PCO) pulp response, or complete pulp healing.

In the presence of an immature root system, teeth will tend to show a normal healing process (no pulp reaction) or, even more often, develop PCO. PN, when present, seems to be influenced by the severity of the extrusion and by the stage of root development, as it is more frequent in teeth with a mature root system. 

Only a weak statistical correlation was found between time to treatment and the development of PCs (PN and PCO) (*p* = 0.060, Table 2).

The analysis of the therapeutic approaches adopted has shown that manual repositioning of the extruded tooth, soon after the trauma, should be the preferred method, even though the orthodontic repositioning technique through the application of light force did not seem to be associated with any prognostic worsening. 

The study confirmed that PN after PCO is a rare event, and that the therapeutic use of orthodontic repositioning does not seem to increase its chances of occurring.

In the future, it will be worth examining a larger sample in order to obtain, through meta-analyses, more reliable data that may facilitate the identification of a significant association between the different parameters considered. 

However, a standardized and repeatable method of data collection, carefully conducted throughout the diagnostic and monitoring phases, may provide further data to be integrated in future studies in the field of traumatic dental injury

## Figures and Tables

**Table 1:** The table shows associations between covariates and pulp consequences (PCO and PN).

Covariate	Association with PC’s	Test Used
mm of extrusion (cat.)	*p*-value = 0.727	Fisher’s exact test
Time to treatment	*p*-value = 0.060 *	Mann–Whitney test
Stage of apical maturation	*p*-value = 0.236	Fisher’s exact test
Type of splinting	*p*-value = 0.345	Fisher’s exact test
Crown color change	*p*-value = 0.318	Fisher’s exact test

* weak evidence (10%) of significant association between time to treatment and Pulp consequences (PCs).

**Table 2:** The table shows epidemiological and etiological data of the sample.

Patient	Gender	Age	Affected Tooth	Etiology	Root Development
**C.**	F	9 years	1.1	Domestic	OA
			2.1	Domestic	OA
**B.**	M	8 years	1.1	Sport	OA
**L.**	M	14 years	1.1	Sport	CA
**N.**	M	16 years	1.1	Assault	CA
**S.**	F	16 years	1.1	Domestic	CA
			2.1	Domestic	CA
**C.**	M	10 years	1.1	Sport	OA
			2.1	Sport	OA
**M.**	M	10 years	1.1	Sport	OA
**P.**	F	8 years	2.1	Domestic	OA
**G.**	F	12 years	2.1	Sport	CA
**S.**	F	9 years	1.1	Sport	OA

**Table 3:** The table shows anamnestic information, treatment performed and clinical pulp consequences.

Patient	Tooth	Apex	Severity of Extrusion	Time to Treatment (Hours)	Treatment	Onset of PCO (Years from Trauma)	Completion of PCO (Years from Trauma)	PN	Sensitivity at Baseline	Sensitivity over Time	Loss of Sensitivity (Time from Trauma)	Crown Color Change (Time from Trauma)	PN after PCO	Other Treatments
**C.**	11	OA	3mm Moderate	8	Manual repositioning and stabilization splinting	1	5	No	Absent	Reduced at 1year	3 years	1–2 years	No	No
	21	OA	2mm Mild	8		1	5	No	Light	Slight	3 years	1–2 years	No	No
**B.**	11	OA	5mm Moderate	10	Orthodontic repositioning	1	5	No	Absent	Reduced at 1year	3 years	Within 1 year	No	No
**L.**	11	CA	3 mm Moderate	10	Manual repositioning	1	5	No	Slight	Reduced at 6 months	3 years	2 years	No	No
**N.**	11	CA	4 mm Moderate	18	Orthodontic repositioning	No PCO	No PCO	Within 1 yr	Slight	Slight	1 year	Within 1 year	No	No
**S.**	21 1.1	CA CA	2 mm Mild 4mm Moderate	10 10	Orthodontic repositioning Orthodontic repositioning	No PCO No PCO	No PCO No PCO	6 months No PN	Slight Present	Slight Present	4 months -	Within 1 year No	No Healed	No No
**C.**	1.1	OA	5 mm Moderate	4	Orthodontic repositioning	1	5	No	Absent	Reduced at 1 year	3 years	2 years	No	No
	2.1	OA	2 mm Mild	4	Orthodontic repositioning	1	5	No	Slight	Slight	3 years	2 years	No	No
**M.**	1.1	OA	6 mm Severe	24	Stabilization splinting	No PCO	No PCO	Yes (within 1yr)	Absent	Absent	Absent	3 years	IRR at 3 years	Started ortho after 1year
**P.**	2.1	OA	2 mm Mild	24	No treatment	1	5	No	Slight	Reduced at 1 year	3 years	3 years	No	No
**G.**	2.1	CA	6 mm Severe	3	Orthodontic repositioning	1	5	No	Absent	Reduced at 1 year	1 year	Within 1 year	No	No
**S.**	1.1	OA	3 mm Moderate	8	Stabilization splinting	1	5	No	Slight	Reduced at 1 year	1 year	1–2 years	No	No

**Table 4:** The table shows the analysis of pulp consequences with respect to covariates (mm of extrusion, type of splinting and time to treatment).

	PCO	PN	Healed
Severity of Extrusion			
Mild extrusion	I I I	I	
Moderate extrusion	I I I I I	I	I
Severe extrusion	I	I	
Total	9	3	1
Type of splinting			
Repositioning splinting	I I I I	I I	I
Stabilization splinting	I I I	I	
No splinting	I I		
Total	9	3	1
Time to treatment			
<4 h	I I I		I
5 to 12 h	I I I I I	I	
13 to 18 h	I	I	
19 to 24 h		I	
Total	9	3	1

**Table 5:** The table shows the analysis of case report data on extrusive luxations.

Authors	Elbay et al. 2014	Ebrahin and Kulkarni 2013	Subay et al. 2007
General information	Twelve years, male, tooth 11. The tooth was treated 20 days post-trauma. Eleven presented open apex, periapical disease, absence of pulp vitality and increased periodontal ligament space without root and bone fracture.	Seven years, male, tooth 12. The tooth was treated 14 days post-trauma. Extrusion of about 5 mm with palatal displacement. Mobility grade III. The tooth was vital and asymptomatic without pathological radiographic signs. Early stage of root development.	Thirteen years, male, tooth 11. The tooth was treated 30 days post-trauma. Mobility grade I, palatal displacement. Presence of premature occlusal contact. The tooth was not vital and radiography showed an increased periodontal ligament space.
Treatment	Orthodontic intrusion and calcium hydroxide apexogenesis	Orthodontic intrusion and debridement of granulation tissue and suture to promote primary wound closure.	Orthodontic intrusion and root canal treatment (CH for 1 week), after which the tooth was filled with gutta-percha.
Duration of treatment	Intrusion was obtained in 3 months. After a further 3 months the tooth was filled with gutta-percha.	Intrusion was obtained in 19 months.	Intrusion was obtained in 4 months with a removable appliance.
Outcomes	Periapical healing, no pathological radiographic or clinical signs.	Continued root development, no pathological radiographic or clinical signs. Normal pulp vitality was conserved.	The tooth appeared normal clinically and radiographically after 1 year.

**Table 6:** The table shows the comparison of pulp consequence data with literature findings.

Samples	Teeth		PCO		PN		PS		PN after PCO	
	N %		N %		N %		N %		N %	
**Andreasen et al., 1987**	48		22	45.83	13	27.08	13	27.08	1	2.08
	31 OA	64.58 OA	19 OA	39.58 OA	2 OA	4.16 OA	10 OA	20.83 OA		
	17 CA	35.42 CA	3 CA	6.25 CA	11 CA	22.92 CA	3 CA	6.25 CA		
Lee et al., 2003 *	55		19	34.54	23	41.81	6	10.90	2	3.63
	24 OA	43.6 OA	16 OA	29.09 OA	11 OA	20.00 OA				
	31 CA	56.36 CA	3 CA	5.45 CA	12CA	21.81CA				
Hecova et al., 2010 **	89		12	13.48	26	29.21	49	55.05	NS	NS
	8 OA	8.99 OA	5 OA	5.61 OA	2 OA	2.24 OA	1 OA	1.12 OA		
	81 CA	91.01 CA	7 CA	7.86 CA	24 CA	26.96 CA	48 CA	53.93 CA		
**Total**	191		53	27.74	62	32.46	62	32.46	3	1.57
	63OA	32.98 OA	40 OA	75.47 OA	15 OA	24.19 OA	11 OA	5.75 OA		
	129CA	67.02 CA	13 CA	24.53 CA	47 CA	75.81 CA	51 CA	26.70 CA		
**Current study**	13		9	69.23	3	23.07	1	7.69	0	0
	8 OA	61.54 OA	7 OA	77.77 OA	1 OA	33.34 OA	0 OA	0 OA		
	5 CA	38.46 CA	2 CA	22.23 CA	2 CA	66.66 CA	1 CA	100 CA		

PCO: pulp canal obliteration; PN: pulp necrosis; PS: pulp survival; NS: not specified. * Lee’s samples: 19 PCO, 23 PN, 3 RR, 1 Lost, 6 PS. ** Hecova’s sample: 12 PCO, 26 PN, 49 PS, 2 IRR, 0 Lost.
